# Supplementary material for: Cryo-electron microscopy structure of the di-domain core of Mycobacterium tuberculosis polyketide synthase 13, essential for mycobacterial mycolic acid synthesis
Source: Microbiology (Reading). 2024 Oct 16;170(10):001505. doi: 10.1099/mic.0.001505 (PMC11649247; doi:10.1099/mic.0.001505)
Supplement: Uncited Supplementary Material 1. [file mic-170-01505-s001.pdf]

Supplementary Information

CryoEM structure of the di-domain core of *Mycobacterium tuberculosis* polyketide synthase 13, essential for mycobacterial mycolic acid synthesis.

Hannah E Johnston[1], Sarah M Batt[1], Alistair K Brown[1,2], Christos Savva[3], Gurdyal S Besra[1]*, Klaus Fütterer[1]*

[1]School of Biosciences and Institute of Microbiology and Infection, University of Birmingham, Birmingham, B15 2TT, UK. [2]Present address: Biosciences Institute, Faculty of Medical Sciences, Newcastle upon Tyne, NE2 4HH, UK. [3]Institute of Structural and Chemical Biology, The University of Leicester, University Road, Leicester, LE1 7RH, United Kingdom. *To whom correspondence should be addressed: G.Besra@bham.ac.uk, K.Futterer@bham.ac.uk.

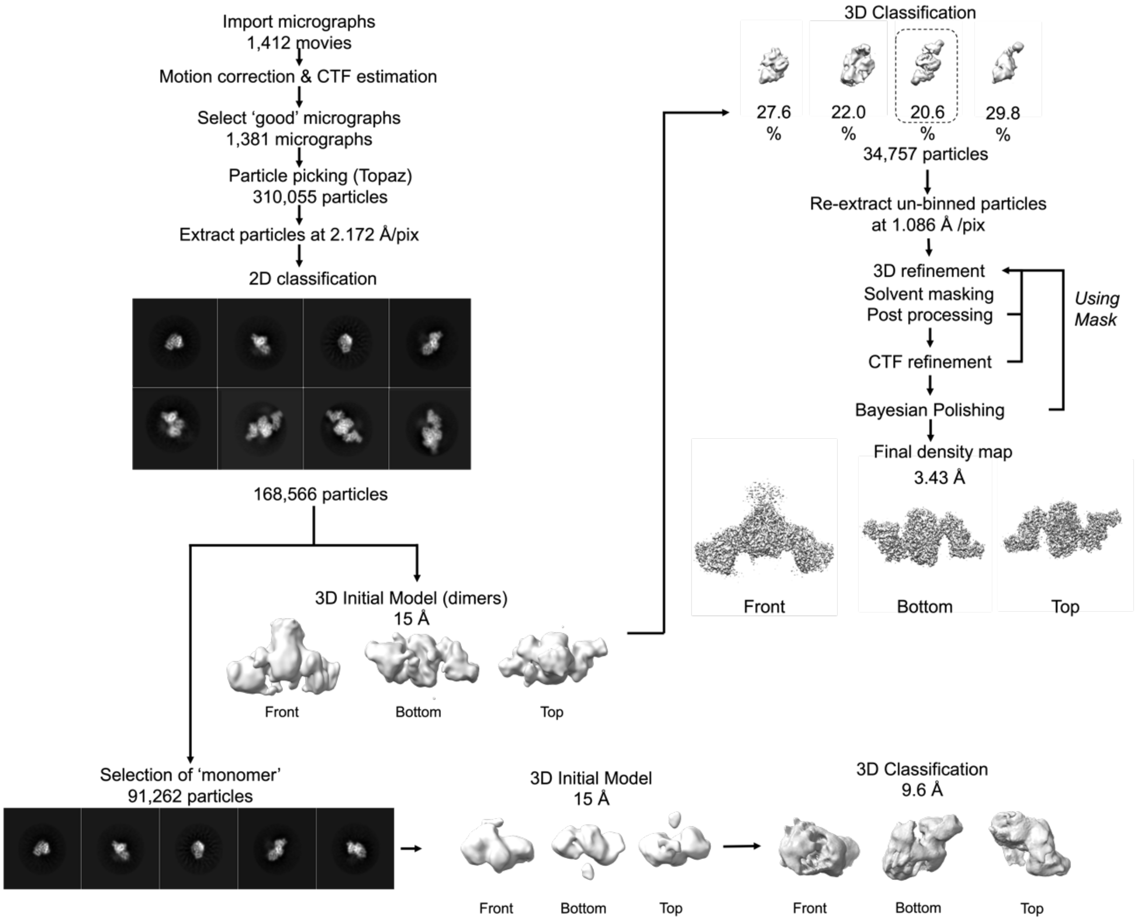

Supplementary Figure 1. CryoEM workflow used to derive the electron density map for Mt-Pks13. Protein was frozen from a storage buffer at pH 7.9 and 2D class averages shows evidence for the presence of a mixed population of monomers and dimers. Only the dimeric particles led to a high-resolution density map (right column), while a low-resolution map could be derived for particles deemed to represent monomers (bottom row).

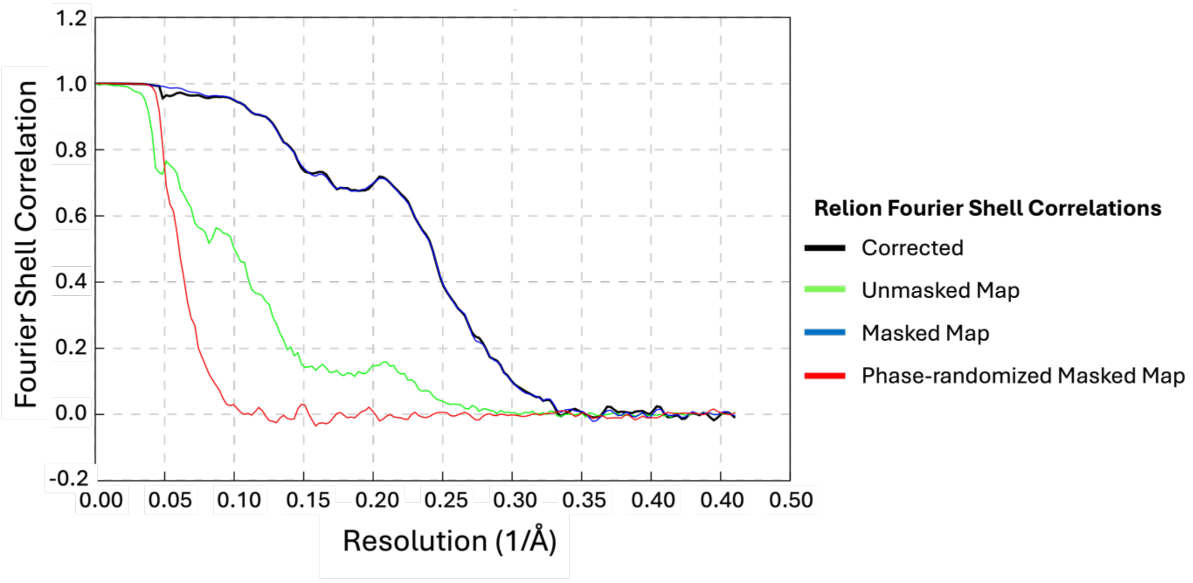

Supplementary Figure 2. Fourier shell correlation vs (inverse) resolution calculated by Relion for the final electron density map.

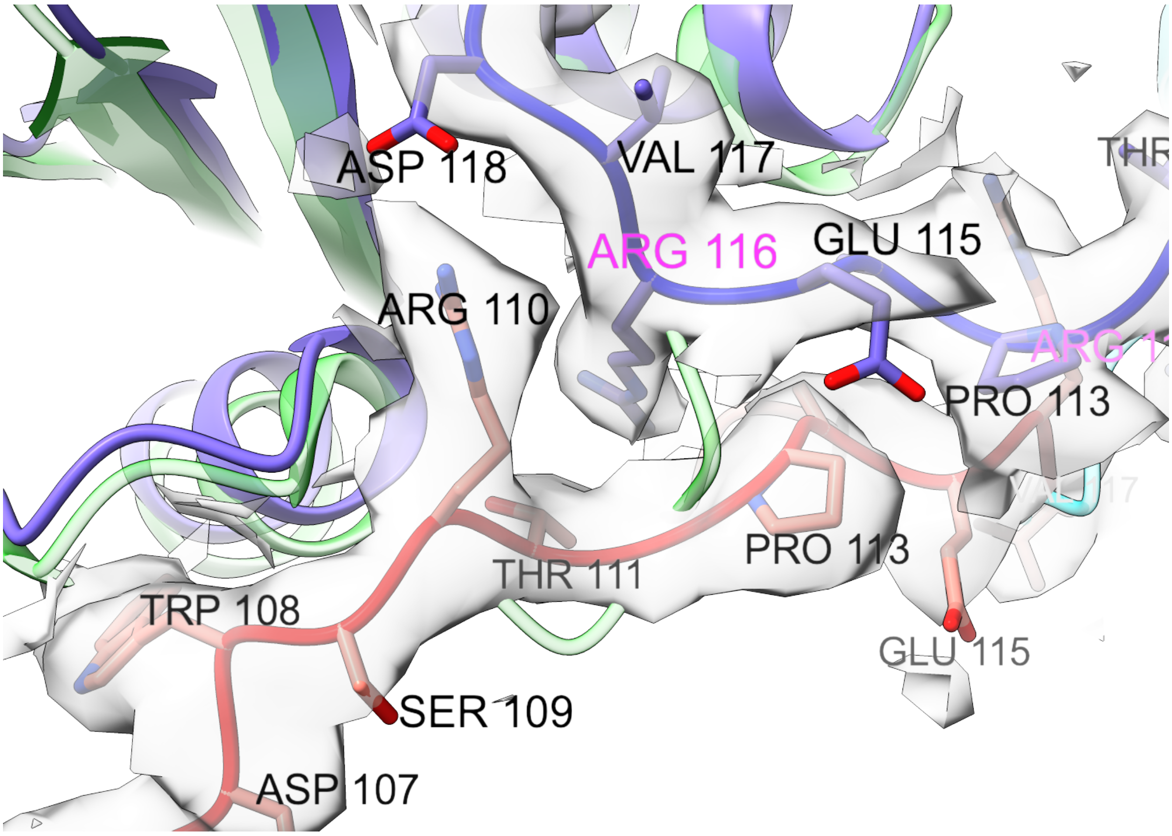

Supplementary Figure 3. Density map for Mt-Pks13 around residue Arg116. Ribbon in green is the Cα trace of Ms-Pks13 (PDB entry 8CV1, (Kim et al., 2023)). Traces in blue and red represent chains A and B, respectively, of Mt-Pks13. Residue numbers refer to the sequence of Mt-Pks13.

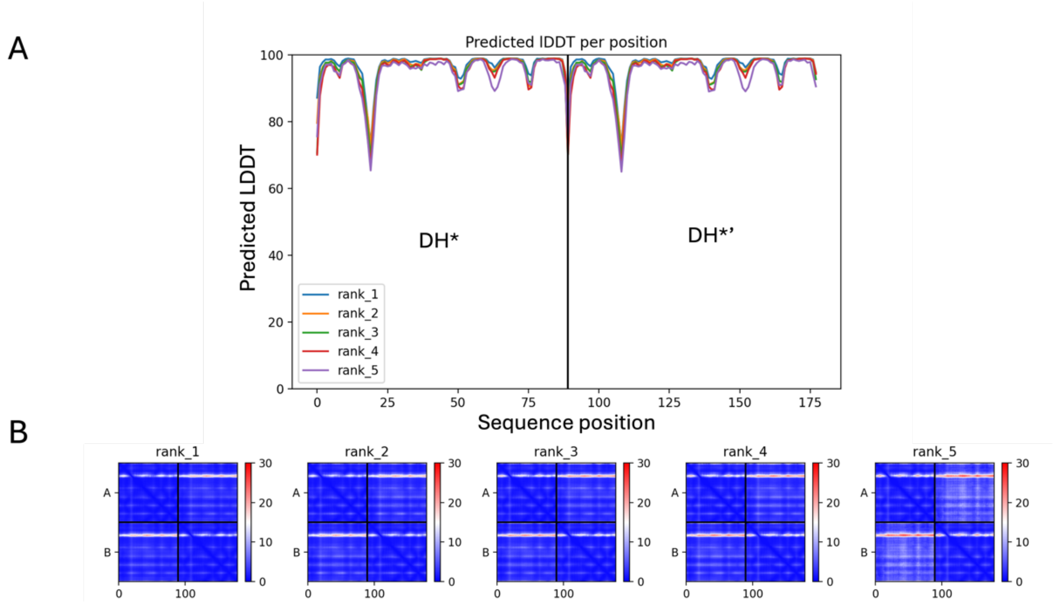

Supplementary Figure 4. Predicted local distance difference test (pLDDT) and predicted aligned error (PAE) plots for the AlphaFold2 prediction of the DH* domain dimer. A pLDDT plot vs sequence position, for the 5 ranked models. B Analogously, PAE plots for models ranked 1 – 5.

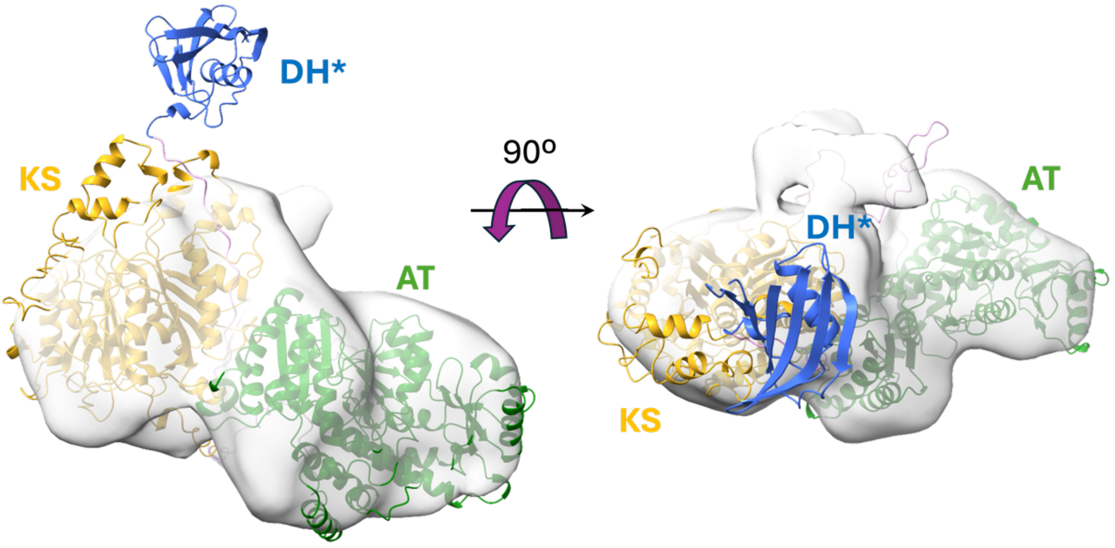

Supplementary Figure 5. Density map for Mt-Pks13 derived from monomeric particles. Front and top views of the electron density map generated from 3D reconstruction of particles deemed to represent monomers (~10 Å resolution, confer Supplementary Fig. 1). Superimposed is a ribbon representation of the AlphaFold2-generated model of the Pks13 fragment comprising residues 105 to 1171, incorporating the KS (gold), AT (green) and DH* (blue) domains.

Supplementary Table 1. Data collection, data processing and structure refinement

Data collection and processing	
Magnification	81000 x
Voltage (kV)	300
Electron exposure (e Å^{-2})	42
Defocus range (μm)	-2.7 to -1.5
Pixel size (Å)	1.086
Symmetry imposed	C1
No. of initial particle images	310,055
No. of final particle images	34,757
Map resolution FSC threshold (Å)	3.43 (FSC=0.14)
Map resolution range (Å)	3.2-13.9
**Refinement**	
Initial model used	3TZZ, 2QO3
Model resolution FSC threshold (Å)	3.43 (FSC=0.143)
Model resolution range (Å)	3.1-12
Map sharpening *B* factor (Å^2)	-52.23
Model composition	
Non-hydrogen atoms	13803
Protein/nucleic acid	1845
Ligands	0
B factors (Å^2)	
Protein	70.54
Ligand	N/A
R.m.s. deviations from ideality	
Bonds (Å)	0.003
Angles (°)	0.689
MolProbity score	2.31
Clashscore	8.24
Poor rotamers (%)	3.15
Ramachandran plot	
Favoured (%)	91.94
Allowed (%)	7.84
Disallowed (%)	0.22

Supplementary Table 2. Analysis of the KS-KS dimer interface using PISA (Krissinel & Henrick, 2007)**.** Listing of H-bond and salt bridge interactions detected by the software.

**H-bond interactions**			
**##**	KS domain (chain A)	Dist. [Å]	KS domain (chain B)
1	A:ARG 116[NH1]	2.40	B:GLY 112[O]
2	A:ALA 114[N]	3.51	B:ALA 114[O]
3	A:ARG 110[NH2]	2.73	B:ASP 118[OD2]
4	A:ARG 387[NH2]	2.65	B:PHE 273[O]
5	A:ARG 387[N]	2.74	B:ASP 275[OD1]
6	A:HIS 384[N]	3.45	B:GLY 278[O]
7	A:ASP 284[N]	3.43	B:THR 282[O]
8	A:THR 282[N]	3.61	B:ASP 284[O]
9	A:SER 264[N]	3.07	B:ASP 284[OD2]
10	A:ARG 116[NH2]	3.53	B:ASN 303[O]
11	A:ARG 405[NH2]	2.76	B:GLU 305[O]
12	A:ARG 110[NH1]	3.12	B:ALA 378[O]
13	A:THR 111[N]	3.21	B:LYS 408[O]
14	A:THR 111[OG1]	2.93	B:LYS 408[O]
15	A:ARG 110[NH1]	2.85	B:ASP 409[O]
16	A:ASP 107[O]	3.38	B:LYS 408[NZ]
17	A:GLY 112[O]	3.22	B:ARG 116[NE]
18	A:ALA 114[O]	3.15	B:ALA 114[N]
19	A:ASP 118[OD1]	3.09	B:ARG 110[NH2]
20	A:PRO 250[O]	2.77	B:ARG 169[NH1]
21	A:ASN 268[O]	3.75	B:HIS 384[NE2]
22	A:TYR 272[O]	3.59	B:ARG 387[NE]
23	A:PHE 273[O]	2.56	B:ARG 387[NH2]
24	A:ASP 275[OD1]	2.67	B:ARG 387[N]
25	A:GLY 278[O]	3.26	B:HIS 384[N]
26	A:THR 282[O]	3.48	B:ASP 284[N]
27	A:ASP 284[O]	3.48	B:THR 282[N]
28	A:ASP 284[OD2]	2.20	B:SER 263[OG]
29	A:GLN 296[OE1]	3.11	B:GLN 296[NE2]
30	A:ASN 303[O]	2.98	B:ARG 116[NH2]
31	A:GLU 305[O]	3.87	B:ARG 405[NH2]
32	A:GLU 305[OE2]	3.29	B:HIS 295[NE2]
33	A:ALA 378[O]	3.12	B:ARG 110[NH1]
34	A:LYS 408[O]	3.37	B:THR 111[OG1]
35	A:LYS 408[O]	3.40	B:THR 111[N]
36	A:ASP 409[O]	3.45	B:ARG 110[NH1]
37	A:ASP 409[OD1]	3.86	B:THR 111[OG1]
**Saltbridge interactions**			
**##**	KS domain (chain A)	Dist. [Å]	KS domain (chain B)
1	A:ARG 110[NH2]	2.73	B:ASP 118[OD2]
2	A:HIS 295[NE2]	3.98	B:GLU 305[OE2]
3	A:ASP 118[OD1]	3.09	B:ARG 110[NH2]
4	A:ASP 118[OD2]	3.93	B:ARG 110[NH1]
5	A:ASP 118[OD2]	3.36	B:ARG 110[NH2]
6	A:GLU 305[OE1]	3.69	B:HIS 295[NE2]
7	A:GLU 305[OE2]	3.29	B:HIS 295[NE2]

Supplementary Table 3. Identifying structural neighbours of the DH* domain using distance matrix alignment as implemented at the DALI server (Holm, 2020). The query structure was the AlphaFold2-generated structural model of the DH* domain of Mt-Pks13 (residues 1072-1171).

No.	Chain	Z	rmsd	lali	nres	%id	Description
1	7zsk-A	11.7	1.9	85	1482	5	PUTATIVE POLYKETIDE SYNTHASE;
2	5il5-B	11	2	84	257	14	MLND;
3	6b2v-A	10.9	1.8	82	267	16	SORB;
4	7vwk-A	10.8	1.9	84	260	14	POLYKETIDE SYNTHASE;
5	3cjy-A	10.8	1.8	81	253	15	PUTATIVE THIOESTERASE;
6	5bp3-A	10.5	1.6	83	286	20	MYCOCEROSIC ACID SYNTHASE-LIKE POLYKETIDE SYNTHASE;
7	3nwz-A	10.2	1.9	83	155	14	BH2602 PROTEIN;
8	3gek-A	10.2	1.8	85	132	12	PUTATIVE THIOESTERASE YHDA;
9	5kku-B	10.1	1.8	79	289	13	POLYKETIDE SYNTHASE TYPE I;
10	4a12-B	10.1	2	83	186	7	TRANSCRIPTION FACTOR FAPR;
11	7cpx-A	10	2.4	89	2262	10	LOVASTATIN NONAKETIDE SYNTHASE, POLYKETIDE SYNTHA
12	3esi-A	9.9	2.2	85	124	6	UNCHARACTERIZED PROTEIN;
13	2prx-A	9.8	1.9	81	114	10	THIOESTERASE SUPERFAMILY PROTEIN;
14	2gvh-B	9.7	2.7	87	250	6	AGR_L_2016P;
15	3kg7-C	9.6	2	85	287	16	CURH;
16	3f1t-B	9.5	2.1	83	137	5	UNCHARACTERIZED PROTEIN Q9I3C8_PSEAE;
17	3oml-A	9.4	2.4	79	532	10	PEROXISOMAL MULTIFUNCTIONAL ENZYME TYPE 2, CG3415
18	1tbu-B	9.4	1.8	76	98	7	PEROXISOMAL ACYL-COENZYME A THIOESTER HYDROLASE
19	4w78-F	9.3	1.9	77	127	6	HYDRATASE CHSH1;
20	3e29-B	9.3	2.1	85	135	16	UNCHARACTERIZED PROTEIN Q7WE92_BORBR;
21	1c8u-A	9.3	2.1	81	285	7	ACYL-COA THIOESTERASE II;
22	3bbj-A	9.2	2	78	268	5	PUTATIVE THIOESTERASE II;
23	3f5o-A	9.1	2.2	86	138	15	THIOESTERASE SUPERFAMILY MEMBER 2;
24	2fs2-B	9.1	2.3	86	138	14	PHENYLACETIC ACID DEGRADATION PROTEIN PAAI;
25	3e8p-A	9.1	2.1	82	153	5	UNCHARACTERIZED PROTEIN;
26	1vi8-B	9	1.9	83	146	5	HYPOTHETICAL PROTEIN YDII;
27	5e1v-B	8.8	2.5	82	274	7	POLYKETIDE SYNTHASE PKSL;
28	3dkz-A	8.8	2.1	81	125	10	THIOESTERASE SUPERFAMILY PROTEIN;
29	2qwz-A	8.8	2.1	86	144	8	PHENYLACETIC ACID DEGRADATION-RELATED PROTEIN;
30	3e1e-C	8.8	2.3	86	141	10	THIOESTERASE FAMILY PROTEIN;

References

Holm, L. (2020). Using Dali for Protein Structure Comparison. In *Methods in Molecular Biology* (Vol. 2112). https://doi.org/10.1007/978-1-0716-0270-6_3

Kim, S. K., Dickinson, M. S., Finer-Moore, J., Guan, Z., Kaake, R. M., Echeverria, I., Chen, J., Pulido, E. H., Sali, A., Krogan, N. J., Rosenberg, O. S., & Stroud, R. M. (2023). Structure and dynamics of the essential endogenous mycobacterial polyketide synthase Pks13. *Nature Structural and Molecular Biology*, *30*(3). https://doi.org/10.1038/s41594-022-00918-0

Krissinel, E., & Henrick, K. (2007). Inference of Macromolecular Assemblies from Crystalline State. *Journal of Molecular Biology*, *372*(3). https://doi.org/10.1016/j.jmb.2007.05.022
